# Heterosexual transmission of HIV in the Dominican Republic: gendered indicators are associated with disparities in condom use

**DOI:** 10.1186/s12889-015-2432-8

**Published:** 2015-11-23

**Authors:** Michelle M. Jimenez, Flavia C. D. Andrade, Marcela Raffaelli, Juliet Iwelunmor

**Affiliations:** Department of Medicine, Pontificia Universidad Catolica Madre y Maestra, Santiago, Dominican Republic; Department of Kinesiology and Community Health, University of Illinois at Urbana-Champaign, Illinois, USA; Department of Human and Community Development, University of Illinois at Urbana-Champaign, Illinois, USA

**Keywords:** HIV risk, Gender differences, Condom use, Heterosexual HIV transmission

## Abstract

**Background:**

Gendered dynamics in heterosexual relationships compromise women’s self-efficacy and increase their vulnerability to acquiring HIV. This study examines the impact of socioeconomic determinants, media exposure, and sexual expectations on sexual behaviors of men and women in the Dominican Republic (DR).

**Methods:**

We analyzed cross-sectional data from 51,018 adults in the Dominican Republic age 15 to 45 years collected by the Demographics and Health Survey (DHS) in 2007. Measures included demographic and socioeconomic indicators, social exposures, sexual expectations and sexual behaviors. Logistic regression models explored gender differences in condom use.

**Results:**

Study findings indicated that women were less likely to use a condom at last intercourse than men (odds ratio [OR] = 0.29; 95 % CI = 0.27, 0.31). Among men, secondary (OR = 1.43; 95 % CI = 1.16, 1.76) and higher education (OR = 1.58; 95 % CI = 1.25, 2.00), being in the richest quintile (OR = 1.25; 95 % CI = 1.07, 1.47), and living in a female-headed household (OR = 1.13; 95 % CI 1.03, 1.23) increased the likelihood of condom use. Compared to never married men, currently and formerly married men were less likely to use condoms (OR = 0.03; 95 % CI = 0.03, 0.04 and OR = 0.67; 95 % CI = 0.60, 0.75, respectively). The odds of condom use increased for young women 15–19 years old in comparison with women age 30–34 years, but decreased as they grew older. For women, being in the richer quintile (OR = 1.28; 95 % CI = 1.06, 1.54), living in a female-headed household (OR = 1.26; 1.12, 1.41), and having good access to media (OR = 1.24; 95 % CI = 1.12, 1.42) increased the likelihood of condom use. Being currently married or formerly married and living in rural areas decreased such likelihood among women.

**Conclusions:**

Study findings provide evidence that, in the DHS, socioeconomic and cultural differences between men and women affects condom use. Efforts to reduce HIV transmission within heterosexual relationships in the DR call for tailored, gender-specific interventions that take into account gender differences of power and sexual behaviors.

**Electronic supplementary material:**

The online version of this article (doi:10.1186/s12889-015-2432-8) contains supplementary material, which is available to authorized users.

## Background

The Dominican Republic (DR) has the second highest number (after Haiti) of adults living with HIV in the Caribbean [1–3]. Over half (56 %) of these HIV cases are women, mainly young women age 15 to 24 [1, 2, 4, 5]. Even though, prevalence rates of HIV infection are similar for men and women, men in DR are much more likely to report having sex with a non-spousal or co-habiting partner, which is considered high risk sex [6]. In fact, DR is rated among the countries with the largest gender disparity in higher risk sex [6]. These differences in sexual behaviors are embedded in a cultural context in which many women in unions in DR are unaware of their male partners’ encounters. As a result, they do not take the necessary steps, such as negotiating condom use, to protect themselves from STDs and HIV [7]. These gender differences in sexual behaviors are particularly problematic in a context in which HIV transmission is primarily heterosexual and mainly attributable to unprotected sex [1, 7, 8].

As in other Caribbean countries, social disparities in HIV rates in the DR have been associated with poverty and limited education. Women with fewer than four years of education—particularly those who live in areas of extreme poverty—are one of the groups most vulnerable to the disease [7, 9]. Low educational level has been found to correlate with lower HIV knowledge, lower risk perception, and more risky sexual behaviors, and therefore correlates with a higher risk of acquiring HIV [7, 10, 11].

In contrast, higher levels of education have been associated with a lower prevalence of HIV in the DR [9]. Previous studies have shown that improving education is effective in reducing HIV risk [12, 13]. Among women, higher education provides them with better job opportunities and financial independence, which some have suggested leads women to be less submissive when negotiating with sexual partners about the use of condoms [14, 15]. However, a higher level of education in women has not always been found to be protective for risky sexual behaviors, such as having multiple sexual partners and inconsistent condom use [16, 17].

Evidence from the DR and other countries indicates that women’s vulnerability to HIV is not solely due to socioeconomic factors (e.g. education, income, occupation, and availability of resources); it is also the outcome of a cumulative history of cultural influences that have shaped specific roles for men and women [18–20]. Regardless of a woman’s level of education, the social normalization of gender roles may explain why she has limited power when communicating about sexuality with her partner, demanding fidelity, seeking self-protection in sexually intimate encounters, and negotiating condom use [14, 15, 19]. In the DR, for instance, women have higher rates of enrollment in secondary and tertiary education, better HIV knowledge, and higher risk perception of acquiring HIV [7, 21]; however, women are less likely to use condoms than men, particularly if they are married or in a committed relationship [7]. This incongruity between what women know and what they do may be explained by culturally driven gender roles [19].

Access to media have the potential to negatively influence gender expectations and roles, but it can also provide information towards adopting good health behaviors, therefore the role of media is complex and depends on exposure and content. For instance, it is not uncommon to find examples in the media that portrays women stereotypically—in subordinate roles, with sexualized bodies, and lacking power in male–female relationships [22, 23], the “cultivation” theory of media effects suggests that media imagery shapes people’s views of social reality, such that those who are higher media consumers (e.g., of TV crime dramas) are more likely to believe the world looks as it does in the media version of reality [24]. For example, previous studies indicate that media coverage about a policy issue (i.e. domestic violence) that highlights a specific target group (i.e. women) can activate stereotypes about that group in individuals’ minds, affecting the way the public (both men and women) thinks about the issue in question [25, 26]. However, media-based interventions targeting HIV risk have been proved to be effective in modeling condom use and condom negotiation, which increases power among women about their own sexuality [21]. This last finding suggest that the role of access to media may also have a positive impact on condom use depending on the type of message given, but limited information exists on this matter.

Several traditional health behavior theories have explained HIV risk through social-cognitive and motivational processes; however, inequalities between men and women within the labor force, in sexual relationships, and through cultural norms and social expectations can lead to women having limited power and lack of control within relationships [11, 15, 20]. The present study uses the Sex, Gender, and Population/Health/Nutrition framework used by the Demographic and Health Survey (DHS) to evaluate the impact of gender differences on sexual behaviors in the DR [27]. The analyses consider how socially constructed differences may determine gender inequalities in roles, responsibilities, expectations, and behaviors, and therefore in power and rights [27]. As a result, the study includes measures related to socioeconomic determinants, media exposure, and sexual expectations, representing sexual norms associated with the culture.

Evidence suggests that in heterosexual relationships, culturally driven gender roles may work together with socioeconomic indicators to exacerbate gendered power-imbalances and lead to sexual behaviors that increase women’s risk of acquiring HIV [15, 19]. Because the main mode of HIV transmission in the DR is heterosexual and is mainly attributable to unprotected sex [1, 7, 8], a comprehensive analysis of gender differences in sexual behaviors needs to include both socioeconomic and cultural factors, and to consider these factors in light of gendered imbalances.

Most of the studies related to HIV risk in the DR have been descriptive [7, 9]—we found no previous studies that examined the predictive associations between sexual behaviors and socioeconomic and cultural factors using a theory-based quantitative analysis focused on gender. Thus, the purpose of the study was to explore the differences in gendered indicators and sexual behaviors between men and women age 15 to 49 years in the DR, and to contrast the impact of gendered indicators on condom use among men and women. Based on the proposed theoretical framework the study hypothesized that (1) higher socioeconomic status increase the likelihood of condom use for both men and women; (2) higher exposure to media may increase condom use, mainly for women; (3) the role of traditional gender sexual expectations may be associated with lower condom use, mainly for women.

## Methods

### Data and selection of participants

We used cross-sectional population data from the DHS that was collected in the DR in 2007 through face-to-face interviews implemented by the Center of Demographic Studies (CESDEM) [28]. This was the most recent available data set at the moment of the study. All DHS datasets are openly available to the public to download and use under the appropriate permission. To download datasets, the authors completed a short registration form. The DHS program send an email to approve the project and to grant the permission to download 2007 DR data. The project was assigned with the number 43578. The original data collection used a two-stage household sample design. The first sampling frame used geographical census areas consisting of households. In the second stage, a fixed number of households were randomly and systematically selected from each area. Among the selected households, all eligible women age 15–49 and men age 15–59 who agreed to participate were interviewed [28].

Our study used data only from men and women age 15–49 who completed their gender-specific questionnaire. The analytic sample consisted of 51,018 participants, of whom 24,106 were men and 26,912 women.

### Measures

#### Demographic, socioeconomic indicators and media exposure

Demographic measures included gender (0 = male, 1 = female) and age. Age was measured using 5-year age group categories (15–19, 20–24, 25–29, 30–34, 35–39, 40–44, and 45–49 years old) according to participants’ self-reported age. The 30–34 age group was used as the reference group because this was the median age.

Socioeconomic characteristics included marital status, education, wealth, and residence. Marital status was categorized as never married (reference group), currently married (married and living together), and formerly married (widowed, divorced, and not living together). Education was categorized as no education (reference group), primary, secondary, and higher, according to participants’ self-reported highest educational achievement. Wealth was measured using DHS wealth quintiles based on the wealth index, which presents the distribution of individual households on a continuous scale of relative wealth—poorest (reference group), poorer, middle, richer, and richest [28, 29]. Residential location was categorized as capital/big city (population over 1 million, reference group), small cities (population over 50,000), towns (other smaller urban areas), and countryside (all rural areas). A binary variable was used to indicate the sex of the household head (0 = male, 1 = female).

Access to media was assessed by asking participants how often they had watched television, read a magazine, or both and was categorized as limited (not at all or less than once a week, reference group), fair (at least once a week), and good (almost every day).

#### Sexual expectations

Gendered sexual expectations refer to culturally driven gender roles and social norms that may shape sexual behaviors [11, 27]. Gendered sexual expectations include abstinence before marriage, sexual exclusiveness, and sexual submissiveness. Respondents selected one of four responses that best reflected their views on whether it was acceptable for young men and women to abstain from sex before marriage. The possible responses were not acceptable for men or women (that is, neither men nor women should wait until marriage to have sex), acceptable for men but not for women, acceptable for women but not for men, and acceptable for both. The third response option, that abstinence was acceptable for women but not for men, was used as the reference group because it favors traditional gender roles.

The same four responses were used for the measure of acceptance of sexual exclusiveness—whether unmarried sexually active men and women should have only one sexual partner at a time, and acceptance of faithfulness to spouse—whether married men and women should have sex only with their spouse or partner. Women’s sexual submissiveness was categorized according to respondents’ answers to the statement that a wife is not justified in asking her husband to use a condom if she knows he has a sexually transmitted disease. The possible responses were agree, disagree (reference group), and do not know/no response.

#### Sexual behaviors

Among respondents who reported sexual activity in the last 12 months, condom use was measured as a binary variable (0 = no, 1 = yes) according to participants’ self-reported condom use during the most recent intercourse. Among those who reported ever having sexual intercourse, premarital sex was computed as a binary variable (0 = no, 1 = yes) based on the difference between age at first intercourse and age at marriage. If age at first intercourse was less than age at marriage, then the participant was considered to have had premarital sex. In contrast, an age at first sexual intercourse that was equal to or greater than age at marriage or at union was considered sexual initiation at or after marriage (reference group). Number of sexual partners, including the spouse, in the last 12 months was categorized as none, only one, two, and three or more. The reference group for number of sexual partners was none; reported values for three or more ranged up to 95 sexual partners.

#### Statistical analysis

We used STATA SE version 12.1 (StataCorp, College Station, TX, USA) to perform data analysis. We examined the descriptive statistics using the STATA survey package with linearization estimation in order to account for the complex DHS survey design features (e.g., weight, stratification, and clustering). We explored frequencies and percentages for categorical variables and calculated means and standard deviations for quantitative variables. Pearson’s design-based adjusted chi-square (F-based design ratio) was calculated to estimate gender differences between proportions across categories.

We used nested logistic regression to estimate the predictive odds of gendered power indicators on condom use. Multicollinearity between independent variables was assessed by using the variance inflation factor (VIF) perturbation approach to diagnose collinearity between categorical variables considered as a set of dummy variables [30]. A VIF greater than ten suggests important collinearity between variables, which may mislead statistical calculations [30]. However, a high VIF can be safely ignored when the high VIF indicator refers to dummy variables that represent a categorical variable with three or more categories. In our data, there were only two high VIF values (highest was 12.6). These values were found in the condom regression for women and found for dummy variables related to categorical variables with three or more categories – marital status and sexual exclusiveness.

We computed four nested models [29]. Model 1 included just demographics. Model 2 added education, wealth, and residential location. Model 3 added head of household and access to media. Model 4, the full model, added abstinence until marriage, sexual exclusiveness, and women’s sexual submissiveness. Selection of the order for the models was guided by the adopted theoretical framework. Data analyses were performed first for the entire sample, then disaggregated by gender.

## Results

A total of 51,018 participants age 15 to 49—47.2 % men and 52.8 % women—were included in the data analysis. Mean age was 29.3 ± 0.9 for men and 29.7 ± 0.8 for women. Sample demographics and gendered indicators (overall and by gender) are shown in Table 1.

**Table 1:** Demographic characteristics and gendered indicators of adults age 15–49 years in the Dominican Republic by gender (DHS 2007)

	Men *n =* 24,106 (47.4 %)		Women *n* = 26,912 (52.5 %)		Total *N* = 51,018	
Age (years)^***^	n	%	n	%	n	%
15–19	5676	23.5	5778	21.5	11,452	21.5
20–24	4034	16.7	4303	16.0	8337	16.5
25–29	3462	14.4	3873	14.4	7335	14.9
30–34	2960	12.3	3707	13.8	6667	13.5
35–40	2817	11.7	3535	13.1	6352	12.6
41–44	2786	11.6	3140	11.7	5926	11.5
45–49	2371	9.8	2576	9.6	4947	9.6
Ever married^***^						
Never	10,003	40.9	6176	24.9	16,179	32.0
Currently	10,940	45.7	15,687	56.6	26,627	51.5
Formerly	3163	13.4	5049	19.4	8212	16.5
Highest level of education completed***						
None	1180	4.9	1184	4.4	2364	3.3
Primary	11,545	47.9	10,992	40.8	22,537	40.3
Secondary	8706	36.1	10,132	37.6	18,838	38.9
Higher	2675	11.1	4604	17.1	7279	17.5
Wealth***						
Poorest	7674	20.9	6553	15.4	14,227	18.0
Poorer	5206	19.7	5874	19.2	11,077	19.5
Middle	4541	20.1	5669	21.1	10,210	20.6
Richer	3858	20.3	4907	21.8	8765	21.1
Richest	2830	19.0	3909	22.5	6739	20.8
Area of Residence						
Capital/large city	1899	30.4	2348	31.5	4236	31.0
Small city	4725	21.2	5806	22.6	10,452	21.9
Town	7255	18.2	8222	17.8	15,348	18.0
Countryside	10,564	30.3	10,819	28.0	20,982	29.1
Head of household***						
Male	18,052	74.9	17,070	63.4	35,122	67.5
Female	6054	25.1	9842	36.6	15,896	32.5
Access to media***						
Limited	4030	16.7	5637	20.9	9667	16.4
Fair	3071	12.7	2901	10.8	5972	11.5
Good	16,987	70.5	18,347	68.2	35,334	72.1
Missing data	18	0.1	27	0.1	45	0.0
Abstinence until marriage^a***^						
Not acceptable for men or women	5488	22.8	2937	10.9	8425	16.9
Acceptable for men, not for women	873	3.6	757	2.8	1630	3.1
Acceptable for women, not for men	6853	28.4	6577	24.4	13,430	26.9
Acceptable for both men and women	10,892	45.2	16,641	61.8	27,533	53.1
Sexual exclusiveness^b***^						
Not acceptable for men or women	3680	13.6	1677	6.0	5357	9.6
Acceptable for men, not for women	1984	7.7	1208	4.2	3192	5.9
Acceptable for women, not for men	3264	12.1	2138	7.6	5402	9.7
Acceptable for both men and women	15,178	66.6	21,889	82.2	37,067	74.8
Women’s sexual submissiveness^c**^						
Disagree	23,325	97.1	26,151	97.2	49,476	97.4
Agree	631	2.2	575	1.7	1206	1.9
Do not know/No response	150	0.6	186	0.6	336	0.6

*DHS* Demographic and Health Surveys. ^a^Abstinence until marriage: participants’ opinion on whether young men/women should wait until marriage to initiate sexual activity. ^b^Sexual exclusiveness: participants’ opinion on whether sexually active unmarried men/women should have sex with only one sexual partner. ^c^Respondents’ answers to the statement that a wife is not justified in asking her husband to use a condom if she knows he has a sexually transmitted disease

*P < 0.10; ** *P* < 0.05; *** *P* < 0.001

Most participants completed primary education (40.3 %); however, a higher proportion of women had complete higher education than men. A greater percentage of women than men was found in the richest category. Regardless of gender, about two thirds of the sample lived either in a capital/large city or in the countryside. More than two thirds of the households were male-headed. Although three quarters of the participants (72.1 %) reported having good access to media, a higher proportion of women had limited access to media than men. More women (61.8 %) than men (45.2 %) agreed that abstinence before marriage was acceptable for both men and women. Most participants agreed that both men and women are expected to practice sexual exclusiveness and disagreed with women’s sexual submissiveness.

### Sexual behaviors

Table 2 displays sexual behaviors related to HIV risk. Among participants who were sexually active in the last 12 months, 77.7 % reported not using a condom in their last sexual encounter. Women failed to use a condom in a greater proportion than men (88.5 % vs. 66.7 %, respectively). Approximately 64.1 % of the participants, mainly men, reported premarital sex. A higher percentage of men than women reported multiple sexual partners (23.7 % vs. 2.5 %, respectively).

**Table 2:** Sexual behaviors among adults 15–49 years in the Dominican Republic by gender (DHS 2007)

Sexual behaviors	Men		Women		Total	
	*n*	%	*n*	%	*n*	%
Condom use^a***^						
No	12,781	66.7	17,900	88.5	30,681	77.7
Yes	6219	33.3	1922	11.5	8141	22.3
Premarital sex^b***^						
No	1876	7.8	14,467	62.2	16,343	35.9
Yes	18,752	92.0	7824	37.8	26,576	64.1
Number of sexual partners^c***^						
None	5031	20.9	6962	25.9	11,993	22.8
Just one sexual partner	13,254	55.0	19,150	71.2	32,404	64
Two sexual partners	4167	17.3	604	2.2	4771	9.7
Three or more	1548	6.4	87	0.3	1635	3.5

*DHS* Demographic and Health Survey

^a^Condom use: Included all participants who had been sexually active in the last 12 months. This sample excluded 12,163 participants (5092 men and 7071 women) who were not sexually active in the last 12 months and 33 cases who did not respond to the question. ^b^Premarital Sex: Included all men and women whose age at marriage was greater than their age at first sexual intercourse despite their sexual activity. The sample excluded 8038 cases that had not initiated sexual intercourse and 61 cases with no response/don not know answers. ^c^Number of sexual partners: Categories include the number of sexual partners including the husband in the last 12 months among all currently and formerly married women; counts ranged from 0 to 95+. The sample excluded 215 participants (106 men and 109 women) who had not heard about AIDS

*P < 0.10; ** *P* < 0.05; ***P < 0.001

### Condom use

Full models (Model 4) for all respondents and disaggregated by gender are displayed in Additional file 1: Table S3. Additional file 1: Tables S4, S5 and S6 display all nested models from 1 through 4 [see Additional file 1]. Just adjusting for gender, women were less likely to use a condom than men (OR = 0.22, 95 % CI = 0.21, 0.23) (Additional file 1: Table S4). Although the likelihood of women using a condom increased when adding demographics (Model 1), it remained lower than men (OR = 0.31, 95 % CI = 0.29, 0.33). When disaggregating by gender, the odds of condom use among women progressively decreased in older age groups in comparison with those age 30–34 years (40–44 years: OR = 0.56, 95 % CI = 0.44, 0.72 and 45–49 years: OR = 0.45, 95 % CI = 0.34, 0.60). Age was not significant among men. In comparison with never married women, being currently married or formerly married decreased the likelihood of condom use (OR = 0.08, 95 % CI = 0.07, 0.10 and OR = 0.63, 95 % CI = 0.54, 0.73, respectively). Similar results were found among men (Additional file 1: Table S3). The impact of marital status on condom use was consistent in the full model for both men and women (Additional file 1: Table S3).

In Model 2, men and women with secondary and post-secondary education were more likely to use a condom than women with no education (Additional file 1: Tables S5 and S6). Model 2 also shows that in comparison those in the poorest quintile, as wealth status rose among men and women, condom use increased (Additional file 1: Tables S5 and S6). Women living in towns and in the countryside were less likely than big-city dwellers to use condoms (OR = 0.84, 95 % CI = 0.70, 1.00 and OR = 0.80, 95 % CI = 0.67, 0.96, respectively). In contrast, men who lived in towns were more likely than those who lived in large cities to use condoms (OR = 1.17, 95 % CI = 1.00, 1.37) (Additional file 1: Table S6).

In Model 3, having a female head of household (vs. a male head) increased the likelihood of condom use among women (OR = 1.26, 95 % CI = 1.12, 1.42) and among men (OR = 1.13, 95 % CI = 1.03, 1.23) (Model 3, Additional file 1: Tables S5 and S6). Although the odds of condom use increased among women who had fair (OR = 1.26, 95 % CI = 1.03, 1.54) and good (OR = 1.24, 95 % CI = 1.07, 1.42) (vs. limited) access to media, respectively (Additional file 1: Table S6), access to media did not have a significant effect on men’s condom use (Additional file 1: Table S6). These findings remain consistent in the full model.

In Model 4, none of the differences in sexual expectations among men had an effect on condom use (Additional file 1: Table S6). Among women for the most part, differences in sexual expectations also did not have a significant effect on condom use (Additional file 1: Table S5). However, women who believed that abstinence before marriage should be acceptable for both men and women were less likely to use condoms (OR = 0.84, 95 % CI = 0.74, 0.96) than women who agreed that sexual abstinence before marriage was acceptable only for women (Additional file 1: Table S5). Women who declared that sexual exclusiveness was acceptable for both men and women were more likely to use condoms (OR = 1.21, 95 % CI = 0.99, 1.48) than women who agreed being only acceptable for women, but not men (Additional file 1: Table S5). Among women, agreement with sexual submissiveness decreased the likelihood of condom use by 34 % (Additional file 1: Table S5).

## Discussion

The impact of gender differences on HIV risk has been shown in quantitative [20] and qualitative [31, 32] research. Our study applied the DHS’s Population/Health/Nutrition conceptual framework to explore how a group of gendered indicators may determine differences in HIV risk.

### Differences in power indicators between men and women

Our findings indicate that, in general, Dominican women were more educated than men. According to the 2013 Gender Gap Index Reports women in the DR achieved secondary and tertiary education in higher rates than men [21]. This is particularly important because previous studies have shown that education can improve HIV/AIDS knowledge, increase self-efficacy, and reduce risky sexual behaviors among women [7, 15]. However, despite their higher levels of education, women have higher levels of unemployment than men [19].

As in other countries in Latin America, most of the participants responding to the survey lived in male-headed households [33]. This is consistent with the 2012 World Bank Economic Report, which stated that up to 80 % of households in Latin America and the Caribbean were headed by a man [34]. In addition, results indicated that access to media was typically good for both men and women, but a higher proportion of men had good access to media. Previous studies have found that women’s access to media continues to be limited in several developing countries, and interventions that promote increasing such access need to be developed [31, 35].

Gendered sexual expectations seemed very similar between men and women in our study; however, when comparing specific answers, a greater proportion of women than men agreed with statements expressing the acceptability for both men and women of abstinence until marriage, and sexual exclusiveness. These results are consistent with previous findings in Latin American cultures, where men are expected to be virile and have multiple sexual partners, while women are expected to wait until marriage to initiate sex, to have only one sexual partner, and to be faithful to that partner [18, 36].

Most men and women in our study disagreed with women’s sexual submissiveness; however, it is important to note that this construct was assessed through responses to only one statement, that a wife is not justified in asking her husband to use a condom if she knows he has a sexually transmitted disease. Sanchez and colleagues (2006) have suggested that gender roles prescribe submissive sexual behaviors that are associated with passive roles during sexual activities and the unlikeliness of sexual negotiation [37]. In contrast, our findings suggest that women may perceive some power over their sexuality under extreme circumstances, such as a partner having a sexually transmitted disease. It would be helpful to develop additional measures to assess women’s submissiveness from a more nuanced perspective.

### Differences in sexual behaviors between men and women

As expected, more men than women in the DR reported premarital sex and more than one sexual partner. This is consistent with a recent study that analyzed data from Latin American countries [38] and another study conducted among Nigerian young adults [39]. In addition, our study indicates important gender differences in rates of condom use at last intercourse. Although condom use was low in general, fewer women than men reported using a condom during their last sexual encounter. The small likelihood of women using a condom has been associated with their lower likelihood to negotiate condom use with their sexual partners [7, 40].

### Association between condom use and gendered indicators

Being a man was found to be a protective factor for condom use. This finding is consistent with prior studies in which men were more likely to use condoms and to be more consistent users than women [41, 42]. As found in previous studies, our results indicate that the likelihood of condom use decreases as age increases, particularly among women, a phenomenon that has also been found in the United States [43]. As women age into their reproductive years, they are more likely to be married and thus to perceive less risk of sexually transmitted diseases, including HIV [44]. In addition, married women in developing countries are more likely to use other forms of contraception, such as female sterilization and intrauterine devices, than they are to use condoms [45–47].

Our study shows that being currently married significantly decreased the likelihood of condom use, particularly among women. Other research supports this—one study showed that married and committed women perceived low or no risk of acquiring HIV, and thus were unlikely to use condoms; this was regardless of education or self-efficacy [48]. Nonetheless, studies indicate that never married women have a higher risk of HIV infection in comparison with married women, particularly in younger age groups [31, 49]. Thus, the association between condom use and marital status is complex. Further research should assess the specific needs of women with different marital statuses in the DR in order to better understand the socioeconomic and cultural factors that empower or disempower these women to seek self-protection in heterosexual relationships. Future findings of this research can help guide tailored prevention efforts among women—to enhance communication with their partners and the disclosure of risk behaviors and to promote safer sexual behaviors, including condom use.

As hypothesized, higher education and wealth were protective factors for condom use. Several studies have found that education ameliorates women’s risk of HIV infection in Latin America and Africa and prepares women to challenge gender roles and cultural scripts that shape their vulnerability to HIV [13–15]. In addition, regardless of gender, schooling has been found to be the most consistent predictor of knowledge about HIV/AIDS and preventive behaviors, such as condom use [50]. Equal access to education might also alleviate some aspects of gender-based power inequalities [13].

Contrary with a previous study [26], our results indicate that having a female head of household increases the likelihood of condom use, mainly among women. There are a number of reasons why this might be the case in Latin America. For one, if female-headed households are defined as those who are missing a principal adult male, then by definition they would contain women who have more autonomy in making decisions over their health, including increasing the likelihood of condom use. In addition, there is ample evidence that suggests that women heads are likely to take on much of the financial, emotional, and physical responsibilities of caregiving and sustaining households hence the need to engage in behaviors that decrease women’s risk of acquiring HIV [51]. Finally while available evidence generally equate female headship with household vulnerability, our data reveal the heterogeneity of female-headed households, bringing attention to variation and complexity their decisions surrounding condom use [51].

In agreement with our hypothesis, good access to media was a protective factor for condom use, particularly among women. Moreover, when access to media was added to the model, the protective role of education and wealth on condom use among women declined. These findings are consistent with a recent study that also used DHS data to examine the association between HIV risk, socioeconomic status, and access to media in 13 Sub-Saharan countries [52]. However, DHS data only provide information about traditional media (television and newspapers); it would be interesting to examine the role of contemporary media technology, such as the Internet, on condom use in the DR. Our results suggest that it is necessary to identify potential ways to harness media to reduce HIV risk among women by promoting behavioral change. For instance, access to safer sex modeling television programs are found to increase condom use and self-reported fidelity [11, 53].

As hypothesized, we anticipated that gendered sexual expectations would significantly decrease condom use among women who agreed with traditional attitudes. For instance, greater acceptance of women’s sexual submissiveness was found to decrease the likelihood of condom use among women. The evidence indicates that a submissive woman tends to feel obligated to fulfill her male partner’s needs and desires regardless of his unfaithfulness or HIV status [15, 54]. In fact, there is growing evidence supports the proposition that social obligations may be an explanation for the high number of women infected by their long-term partners, the inconsistent use of condoms among married women, and the challenge of negotiating condom use and refusing unprotected sex among women in formal relationships [14, 15, 55]. Further analyses are needed to better understand the role of HIV-related attitudes about condom use in the DR.

### Implications

According to the literature, the DR has succeeded in slowing HIV transmission, particularly through changes in sexual behavior. For instance, condom use has increased and the number of partners has been reduced among men [9]. Evidence suggests that although HIV prevalence has declined [9]; the number of people living with HIV in the DR continues to be higher among women, whereas condom use continues to be low, particularly among women [1, 2]. Our study illustrates the presence of gender imbalances across rights, responsibilities, and expectations and their association with condom use, leading us to believe that gender imbalances may have an impact on HIV risk. Thus, gender differences in power and sexual behaviors among the Dominican population call for tailored, gender-specific interventions.

Our study points to the protective role of higher education in influencing health behaviors, particularly condom use. However, most Dominicans attain only a primary level of education, with just a small proportion achieving post-secondary education. Thus, our study suggests that improving education should be a goal for the Dominican population regardless of gender. Such interventions may require policy changes and educational reform as well as multi-institutional partnerships. Increasing access to universal education may overcome gendered power-imbalances by providing women with better job opportunities, more financial independence, increased critical thinking and decision making abilities, and skills to negotiate self-protection in their sexual practices [52, 56, 57]. Consequences of education may also include a reduction in rates of poverty and unemployment [52], which, according to our study, negatively impact condom use among women.

The mainly heterosexual nature of the disease in the DR along with high risk sexual behaviors among men, such as multiple sexual partners, makes women vulnerable to be engaged in unsafe sexual practices [9, 45]. Therefore, couple-oriented intervention strategies are needed to facilitate communication and disclosure of risk behaviors as a way to promote safer sexual behaviors among couples.

### Limitations

Results need to be considered in light of study limitations. First, DHS data are cross-sectional; thus, it is not possible to determine causality between gendered power indicators and condom use but instead only to describe the associations [32]. Second, the self-reported nature of the data increases the possibility of bias due to under-reporting of specific sexual behaviors that are perceived not to be socially acceptable, such as young age of first sexual intercourse for women, and bias due to over-reporting of desirable behaviors, such as condom use [47]. However, DHS data have been found to provide nationally representative, reliable estimates [23]. In addition, to account for underrepresentation of some groups due to sample selection, the DHS data used provides information on weight, stratification, and clustering during sampling, thus, data was analyzed using survey features and should provide more accurate estimates [58]. Furthermore, measuring condom use at last intercourse with a single question may result in over- or under-estimates of actual condom use. A recent study suggested that in order to have more accurate estimates of condom use, follow-up questions need to be asked about incomplete condom use and condom use failure [59]. Also, because this question refers to male condom use, women may assume that they are not the ones using the condom, but their male partners. This may underestimate the actual report of condom use among women and explain some of the disparities that the study found. However, previous research has successfully used this question to estimate condom use in several countries in the developing world [29, 60, 61]. Future studies should consider these suggestions to improve primary data collection on condom use, and perhaps other sexual behaviors.

## Conclusions

In conclusion, gender differences in sexual behaviors directly impact the risk of acquiring HIV, particularly among Dominican women. But as women in the DR start to challenge some of the traditional roles by achieving higher levels of education, participating more actively in the work force, and becoming more successful heads of households, use of condoms and other protective sexual behaviors are expected to increase [62–64].

## Additional file

Additional file 1:
**Tables for nested logistic models.** The additional file 1 is an Excel document which contains three separate sheets, each one with a different table that displays the results of the nested analyses for condom use: Table S5 displays the nested logistic models for condom use among all Dominicans age 15 to 45 years in the sample. Table S6 displays the disaggregated nested logistic models for condom use by men. Table S5 displays the disaggregated nested logistic models for condom use by women. (XLSX 37 kb)
